# 2-Methyl­sulfan­yl-1*H*-perimidin-3-ium iodide

**DOI:** 10.1107/S1600536812033697

**Published:** 2012-08-01

**Authors:** Mohammad Hassan Ghorbani

**Affiliations:** a Falavarjan Branch, Islamic Azad University, Falavarjan, Isfahan, Iran

## Abstract

In the structure of the title salt C_12_H_11_N_2_S^+^·I^−^, the methyl­sulfanyl group of the cation is nearly coplanar with the perimidine rings, as indicated by the C—S—C—N torsion angles of 2.9 (5) and −177.2 (3)°, respectively. The (S)C—N bond lengths in the heterocyclic ring are approximately equal [1.325 (5) and 1.326 (6) Å] suggesting a degree of delocalization. In the crystal, cations and anions are linked *via* two discrete N—H⋯I hydrogen bonds, forming chains along the *b* axis.

## Related literature

For synthetic details and applications, see: Liu & Chen (1984 ▶); Herbert *et al.* (1987 ▶). For the NMR spectra, see Woodgate *et al.* (1988 ▶). For related structures, see: Molčanov *et al.* (2012 ▶); Wang (2012 ▶); Tiritiris & Kantlehner (2012 ▶).
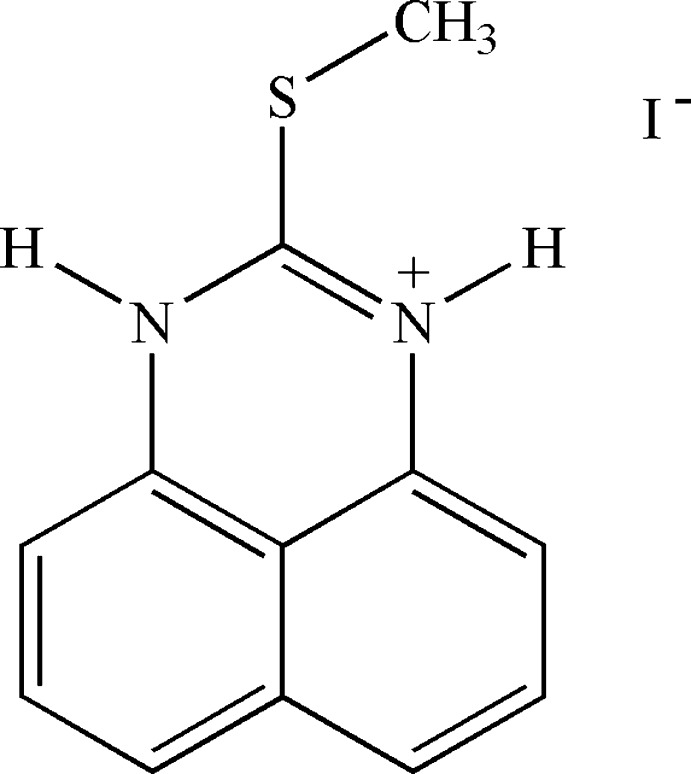


## Experimental

### 

#### Crystal data

C_12_H_11_N_2_S^+^·I^−^
*M* *_r_* = 342.19
Monoclinic, 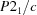

*a* = 7.0107 (14) Å
*b* = 8.8968 (18) Å
*c* = 19.520 (4) Å
β = 95.90 (3)°
*V* = 1211.1 (4) Å^3^
*Z* = 4
Mo *K*α radiation
μ = 2.79 mm^−1^
*T* = 298 K
0.20 × 0.17 × 0.10 mm

#### Data collection

Stoe IPDS 2T diffractometer
Absorption correction: numerical (*X-RED32*; Stoe & Cie, 2005 ▶) *T*_min_ = 0.605, *T*_max_ = 0.768
8695 measured reflections
3266 independent reflections
2151 reflections with *I* > 2σ(*I*)
*R*_int_ = 0.053

#### Refinement

*R*[*F* ^2^ > 2σ(*F* ^2^)] = 0.044
*wR*(*F* ^2^) = 0.094
*S* = 1.11
3266 reflections
152 parameters
1 restraint
H atoms treated by a mixture of independent and constrained refinement
Δρ_max_ = 0.98 e Å^−3^
Δρ_min_ = −0.47 e Å^−3^

### 

Data collection: *X-AREA* (Stoe & Cie, 2005 ▶); cell refinement: *X-AREA*; data reduction: *X-RED32* (Stoe & Cie, 2005 ▶); program(s) used to solve structure: *SHELXS97* (Sheldrick, 2008 ▶); program(s) used to refine structure: *SHELXL97* (Sheldrick, 2008 ▶); molecular graphics: *ORTEP-3 for Windows* (Farrugia, 1997 ▶); software used to prepare material for publication: *WinGX* (Farrugia, 1999 ▶).

## Supplementary Material

Crystal structure: contains datablock(s) I, global. DOI: 10.1107/S1600536812033697/sj5260sup1.cif

Structure factors: contains datablock(s) I. DOI: 10.1107/S1600536812033697/sj5260Isup2.hkl

Supplementary material file. DOI: 10.1107/S1600536812033697/sj5260Isup3.cml

Additional supplementary materials:  crystallographic information; 3D view; checkCIF report

## Figures and Tables

**Table 1:** Hydrogen-bond geometry (Å, °)

*D*—H⋯*A*	*D*—H	H⋯*A*	*D*⋯*A*	*D*—H⋯*A*
N2—H2⋯I1^i^	0.81 (2)	2.72 (3)	3.500 (4)	161 (5)
N1—H1⋯I1	0.85 (6)	2.98 (6)	3.813 (4)	169 (5)

Symmetry code: (i) 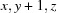.

**Fig. 1.**
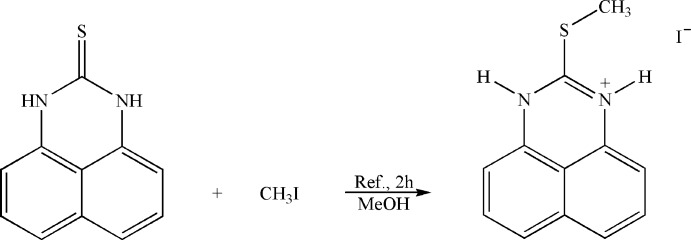
Preparation of the title compound from the reaction of perimidine-2-thione with methyl iodide under reflux conditions.

**Fig. 2.**
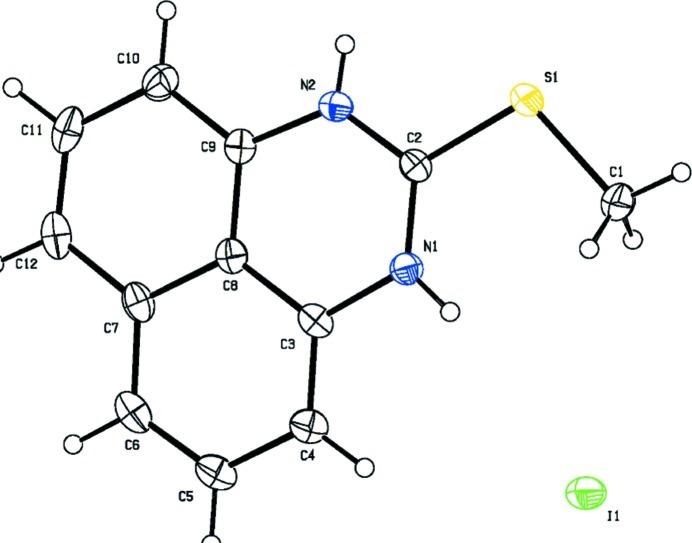
The molecular structure of the title compound, showing 50% probability displacement ellipsoids.

**Fig. 3.**
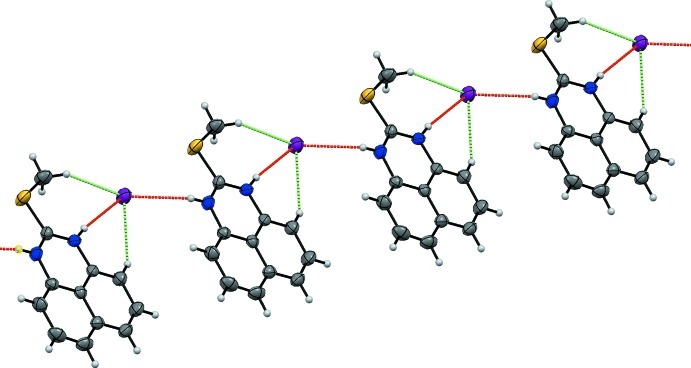
Chains of molecules along the b axis.

## supplementary crystallographic information

### Comment

The title compound is used in the synthesis of some potentially active antitumor agents (Herbert *et al.*, 1987) and heterocyclic compounds (Liu & Chen, 1984). So far, the structure of this compound and its neutral form has been studied using ^13^C and ^1^H NMR spectroscopy (Woodgate *et al.*, 1988). Herein, the crystal structure of this salt is investigated using X-ray crystallography.

In the structure of the 2-methylsulfanylperimidinium cation, the methylsulfanyl group is nearly coplanar with perimidine rings [the torsion angles N1—C2—S—C1 and N2—C2—S—C1 are 2.9 (5)° and -177.2 (3)°, respectively]. Because of conjugation between the lone pair electrons of the S atom and the amidinum moiety (HN—C=NH^+^) in the cation, the C2—S bond length [1.730 (5) Å] is shorter than C1—S [1.789 (5) Å]. Also, like other amidinium cations (Molčanov *et al.*, 2012; Wang, 2012; Tiritiris & Kantlehner, 2012), the C—N bond lengths in the cation are approximately equal [the bond lengths of C2—N1 and C2—N2 are 1.325 (5) Å and 1.325 (6) Å, respectively].

In the crystal lattice, the cations and anions are linked together *via* two different N—H···I hydrogen bonds, in which every iodide anion act as a bridge between two 2-methylsulfanylperimidinium cations.

### Experimental

The title salt was prepared by a literature method (Liu & Chen, 1984; Herbert *et al.*, 1987). Suitable single crystals for X-ray analysis were obtained from ethanol solution at room temperature.

### Refinement

All hydrogen atoms bound to carbon were positioned geometrically with C—H distances = 0.93–0.96 Å and included in a riding model approximation with *U*_iso_(H) = 1.2 or 1.5*U*_eq_(C). The N–H hydrogen atoms were located in a difference Fourier map and refined freely.

### Crystal data

**Table:**

C_12_H_11_N_2_S^+^·I^−^	*F*(000) = 664
*M**_r_* = 342.19	*D*_x_ = 1.877 Mg m^−^^3^
Monoclinic, *P*2_1_/*c*	Mo *K*α radiation, λ = 0.71073 Å
Hall symbol: -P 2ybc	Cell parameters from 3266 reflections
*a* = 7.0107 (14) Å	θ = 2.1–29.2°
*b* = 8.8968 (18) Å	µ = 2.79 mm^−^^1^
*c* = 19.520 (4) Å	*T* = 298 K
β = 95.90 (3)°	Plate, green
*V* = 1211.1 (4) Å^3^	0.20 × 0.17 × 0.10 mm
*Z* = 4	

### Data collection

**Table:**

Stoe IPDS 2T diffractometer	3266 independent reflections
Radiation source: fine-focus sealed tube	2151 reflections with *I* > 2σ(*I*)
Graphite monochromator	*R*_int_ = 0.053
Detector resolution: 0.15 pixels mm^-1^	θ_max_ = 29.2°, θ_min_ = 2.1°
rotation method scans	*h* = −9→9
Absorption correction: numerical (*X-RED32*; Stoe & Cie, 2005)	*k* = −12→10
*T*_min_ = 0.605, *T*_max_ = 0.768	*l* = −26→23
8695 measured reflections	

### Refinement

**Table:**

Refinement on *F*^2^	Primary atom site location: structure-invariant direct methods
Least-squares matrix: full	Secondary atom site location: difference Fourier map
*R*[*F*^2^ > 2σ(*F*^2^)] = 0.044	Hydrogen site location: inferred from neighbouring sites
*wR*(*F*^2^) = 0.094	H atoms treated by a mixture of independent and constrained refinement
*S* = 1.11	*w* = 1/[σ^2^(*F*_o_^2^) + (0.0351*P*)^2^ + 0.0203*P*] where *P* = (*F*_o_^2^ + 2*F*_c_^2^)/3
3266 reflections	(Δ/σ)_max_ = 0.001
152 parameters	Δρ_max_ = 0.98 e Å^−^^3^
1 restraint	Δρ_min_ = −0.47 e Å^−^^3^

### Special details

**Table:**

Geometry. All e.s.d.'s (except the e.s.d. in the dihedral angle between two l.s. planes) are estimated using the full covariance matrix. The cell e.s.d.'s are taken into account individually in the estimation of e.s.d.'s in distances, angles and torsion angles; correlations between e.s.d.'s in cell parameters are only used when they are defined by crystal symmetry. An approximate (isotropic) treatment of cell e.s.d.'s is used for estimating e.s.d.'s involving l.s. planes.
Refinement. Refinement of *F*^2^ against ALL reflections. The weighted *R*-factor *wR* and goodness of fit *S* are based on *F*^2^, conventional *R*-factors *R* are based on *F*, with *F* set to zero for negative *F*^2^. The threshold expression of *F*^2^ > σ(*F*^2^) is used only for calculating *R*-factors(gt) *etc*. and is not relevant to the choice of reflections for refinement. *R*-factors based on *F*^2^ are statistically about twice as large as those based on *F*, and *R*- factors based on ALL data will be even larger.

### Fractional atomic coordinates and isotropic or equivalent isotropic displacement parameters (Å^2^)

**Table:**

	*x*	*y*	*z*	*U*_iso_*/*U*_eq_	
I1	0.23717 (5)	0.01191 (3)	0.335840 (18)	0.04724 (12)	
S1	0.3337 (2)	0.57350 (14)	0.29413 (7)	0.0475 (3)	
N1	0.2710 (5)	0.4113 (4)	0.4069 (2)	0.0308 (8)	
N2	0.3149 (6)	0.6660 (4)	0.4194 (2)	0.0341 (9)	
C1	0.2992 (8)	0.3909 (6)	0.2564 (3)	0.0481 (13)	
H1A	0.3861	0.3211	0.2806	0.072*	
H1B	0.3232	0.3950	0.2089	0.072*	
H1C	0.1696	0.3588	0.2595	0.072*	
C2	0.3031 (6)	0.5445 (5)	0.3799 (2)	0.0304 (9)	
C3	0.2384 (6)	0.3919 (5)	0.4769 (2)	0.0295 (9)	
C4	0.1976 (7)	0.2561 (5)	0.5032 (3)	0.0376 (11)	
H4	0.1910	0.1707	0.4756	0.045*	
C5	0.1651 (7)	0.2459 (6)	0.5733 (3)	0.0432 (12)	
H5	0.1363	0.1531	0.5916	0.052*	
C6	0.1753 (7)	0.3702 (6)	0.6146 (3)	0.0414 (12)	
H6	0.1535	0.3607	0.6606	0.050*	
C7	0.2184 (6)	0.5129 (5)	0.5885 (2)	0.0344 (9)	
C8	0.2492 (5)	0.5236 (5)	0.5181 (2)	0.0295 (8)	
C9	0.2892 (6)	0.6642 (5)	0.4898 (2)	0.0299 (9)	
C10	0.3011 (7)	0.7923 (5)	0.5285 (3)	0.0406 (11)	
H10	0.3295	0.8844	0.5095	0.049*	
C11	0.2689 (7)	0.7801 (6)	0.5986 (3)	0.0451 (13)	
H11	0.2756	0.8664	0.6257	0.054*	
C12	0.2284 (7)	0.6463 (6)	0.6281 (3)	0.0434 (12)	
H12	0.2074	0.6427	0.6744	0.052*	
H1	0.274 (8)	0.328 (7)	0.387 (3)	0.052*	
H2	0.327 (8)	0.746 (4)	0.401 (3)	0.052*	

### Atomic displacement parameters (Å^2^)

**Table:**

	*U*^11^	*U*^22^	*U*^33^	*U*^12^	*U*^13^	*U*^23^
I1	0.0704 (2)	0.02971 (16)	0.04296 (19)	−0.00194 (17)	0.01260 (14)	0.00215 (16)
S1	0.0825 (10)	0.0347 (6)	0.0260 (6)	−0.0092 (6)	0.0095 (6)	0.0024 (5)
N1	0.036 (2)	0.0292 (18)	0.029 (2)	−0.0009 (15)	0.0099 (16)	−0.0010 (16)
N2	0.046 (2)	0.0261 (18)	0.031 (2)	−0.0010 (16)	0.0056 (18)	0.0030 (15)
C1	0.074 (4)	0.037 (3)	0.034 (3)	−0.008 (2)	0.011 (3)	−0.008 (2)
C2	0.031 (2)	0.032 (2)	0.028 (2)	0.0019 (17)	0.0010 (18)	0.0031 (17)
C3	0.025 (2)	0.035 (2)	0.030 (2)	0.0053 (17)	0.0057 (18)	0.0056 (18)
C4	0.038 (3)	0.036 (2)	0.039 (3)	0.000 (2)	0.007 (2)	0.004 (2)
C5	0.047 (3)	0.044 (3)	0.040 (3)	−0.001 (2)	0.011 (2)	0.011 (2)
C6	0.038 (3)	0.056 (3)	0.030 (3)	0.005 (2)	0.006 (2)	0.009 (2)
C7	0.0287 (19)	0.047 (3)	0.027 (2)	0.006 (2)	0.0014 (16)	0.002 (2)
C8	0.0248 (18)	0.034 (2)	0.029 (2)	0.0038 (17)	0.0010 (16)	−0.0004 (19)
C9	0.027 (2)	0.033 (2)	0.029 (2)	0.0021 (17)	0.0024 (18)	−0.0025 (18)
C10	0.047 (3)	0.033 (2)	0.040 (3)	0.003 (2)	0.003 (2)	−0.005 (2)
C11	0.046 (3)	0.048 (3)	0.040 (3)	0.003 (2)	−0.002 (2)	−0.019 (2)
C12	0.041 (3)	0.062 (3)	0.026 (3)	0.004 (2)	0.001 (2)	−0.007 (2)

### Geometric parameters (Å, º)

**Table:**

S1—C2	1.730 (5)	C4—H4	0.9300
S1—C1	1.789 (5)	C5—C6	1.366 (7)
N1—C2	1.325 (5)	C5—H5	0.9300
N1—C3	1.418 (6)	C6—C7	1.413 (7)
N1—H1	0.85 (6)	C6—H6	0.9300
N2—C2	1.325 (6)	C7—C12	1.415 (7)
N2—C9	1.404 (6)	C7—C8	1.415 (6)
N2—H2	0.811 (19)	C8—C9	1.408 (6)
C1—H1A	0.9600	C9—C10	1.366 (6)
C1—H1B	0.9600	C10—C11	1.414 (7)
C1—H1C	0.9600	C10—H10	0.9300
C3—C4	1.356 (6)	C11—C12	1.365 (8)
C3—C8	1.419 (6)	C11—H11	0.9300
C4—C5	1.412 (7)	C12—H12	0.9300
			
C2—S1—C1	103.8 (2)	C6—C5—H5	119.5
C2—N1—C3	122.9 (4)	C4—C5—H5	119.5
C2—N1—H1	126 (4)	C5—C6—C7	121.0 (4)
C3—N1—H1	111 (4)	C5—C6—H6	119.5
C2—N2—C9	123.6 (4)	C7—C6—H6	119.5
C2—N2—H2	118 (4)	C6—C7—C12	123.8 (4)
C9—N2—H2	119 (4)	C6—C7—C8	118.0 (4)
S1—C1—H1A	109.5	C12—C7—C8	118.1 (4)
S1—C1—H1B	109.5	C9—C8—C7	119.8 (4)
H1A—C1—H1B	109.5	C9—C8—C3	120.8 (4)
S1—C1—H1C	109.5	C7—C8—C3	119.4 (4)
H1A—C1—H1C	109.5	C10—C9—N2	121.7 (4)
H1B—C1—H1C	109.5	C10—C9—C8	121.8 (4)
N1—C2—N2	120.1 (4)	N2—C9—C8	116.5 (4)
N1—C2—S1	124.2 (3)	C9—C10—C11	117.7 (5)
N2—C2—S1	115.8 (3)	C9—C10—H10	121.1
C4—C3—N1	122.4 (4)	C11—C10—H10	121.1
C4—C3—C8	121.4 (4)	C12—C11—C10	122.4 (5)
N1—C3—C8	116.2 (4)	C12—C11—H11	118.8
C3—C4—C5	119.1 (5)	C10—C11—H11	118.8
C3—C4—H4	120.4	C11—C12—C7	120.1 (5)
C5—C4—H4	120.4	C11—C12—H12	119.9
C6—C5—C4	121.0 (5)	C7—C12—H12	119.9
			
C3—N1—C2—N2	3.3 (7)	C12—C7—C8—C3	−179.5 (4)
C3—N1—C2—S1	−177.2 (3)	C4—C3—C8—C9	−179.2 (4)
C9—N2—C2—N1	−2.1 (7)	N1—C3—C8—C9	0.3 (6)
C9—N2—C2—S1	178.4 (3)	C4—C3—C8—C7	0.6 (6)
C1—S1—C2—N1	2.9 (5)	N1—C3—C8—C7	−179.9 (4)
C1—S1—C2—N2	−177.7 (4)	C2—N2—C9—C10	−179.5 (4)
C2—N1—C3—C4	177.1 (4)	C2—N2—C9—C8	0.0 (6)
C2—N1—C3—C8	−2.4 (6)	C7—C8—C9—C10	0.5 (6)
N1—C3—C4—C5	−179.4 (4)	C3—C8—C9—C10	−179.7 (4)
C8—C3—C4—C5	0.0 (7)	C7—C8—C9—N2	−179.0 (4)
C3—C4—C5—C6	−0.4 (8)	C3—C8—C9—N2	0.8 (6)
C4—C5—C6—C7	0.1 (8)	N2—C9—C10—C11	178.6 (5)
C5—C6—C7—C12	179.1 (5)	C8—C9—C10—C11	−0.9 (7)
C5—C6—C7—C8	0.5 (7)	C9—C10—C11—C12	0.5 (8)
C6—C7—C8—C9	178.9 (4)	C10—C11—C12—C7	0.3 (8)
C12—C7—C8—C9	0.3 (6)	C6—C7—C12—C11	−179.3 (5)
C6—C7—C8—C3	−0.8 (6)	C8—C7—C12—C11	−0.7 (7)

### Hydrogen-bond geometry (Å, º)

**Table:**

*D*—H···*A*	*D*—H	H···*A*	*D*···*A*	*D*—H···*A*
N2—H2···I1^i^	0.81 (2)	2.72 (3)	3.500 (4)	161 (5)
N1—H1···I1	0.85 (6)	2.98 (6)	3.813 (4)	169 (5)

Symmetry code: (i) *x*, *y*+1, *z*.
